# Prognostic roles for fibroblast growth factor receptor family members in malignant peripheral nerve sheath tumor

**DOI:** 10.18632/oncotarget.8067

**Published:** 2016-03-14

**Authors:** Wenya Zhou, Xiaoling Du, Fengju Song, Hong Zheng, Kexin Chen, Wei Zhang, Jilong Yang

**Affiliations:** ^1^ Department of Bone and Soft Tissue Tumor and Tianjin Medical University Cancer Institute and Hospital, Tianjin 300060, People's Republic of China; ^2^ National Clinical Research Center of Cancer, Tianjin Medical University Cancer Institute and Hospital, Tianjin 300060, People's Republic of China; ^3^ Department of Diagnostics, Tianjin Medical University, Tianjin 300061, People's Republic of China; ^4^ Department of Epidemiology and Biostatistics, Tianjin Medical University Cancer Institute and Hospital, Tianjin 300060, People's Republic of China; ^5^ Department of Pathology, The University of Texas MD Anderson Cancer Center, Houston, TX, 77030 USA

**Keywords:** malignant peripheral nerve sheath tumor, fibroblast growth factor receptor, prognosis, microarray-based comparative genomic hybridization, fluorescence in situ hybridization

## Abstract

**Background:**

Malignant peripheral nerve sheath tumors (MPNST) are rare, highly malignant, and poorly understood sarcomas. The often poor outcome of MPNST highlights the necessity of identifying prognostic predictors for this aggressive sarcoma. Here, we investigate the role of fibroblast growth factor receptor (FGFR) family members in human MPNSTs.

**Results:**

aCGH and bioinformatics analysis identified frequent amplification of the *FGFR1* gene. FISH analysis revealed that 26.9% MPNST samples had amplification of *FGFR1*, with both focal and polysomy patterns observed. IHC identified that FGFR1 protein expression was positively correlated with *FGFR1* gene amplification. High expression of FGFR1 protein was associated with better overall survival (OS) and was an independent prognostic predictor for OS of MPNST patients. Additionally, combined expression of FGFR1 and FGFR2 protein characterized a subtype of MPNST with better OS. FGFR4 protein was expressed 82.3% of MPNST samples, and was associated with poor disease-free survival.

**Materials and Methods:**

We performed microarray-based comparative genomic hybridization (aCGH) profiling of two cohorts of primary MPNST tissue samples including 25 patients treated at The University of Texas MD Anderson Cancer Center and 26 patients from Tianjin Medical University Cancer Institute and Hospital. Fluorescence *in situ* hybridization (FISH) was used to validate the gene amplification detected by aCGH analysis. Another cohort of 63 formalin-fixed paraffin-embedded MPNST samples (including 52 samples for FISH assay) was obtained to explore FGFR1, 2, 3, and 4 protein expression by immunohistochemical (IHC) analysis.

**Conclusions:**

Our integrated genomic and molecular studies provide evidence that FGFRs play different prognostic roles in MPNST.

## INTRODUCTION

Malignant peripheral nerve sheath tumors (MPNSTs) are highly malignant sarcomas derived from the neural crest and account for approximately 5–10% of all soft tissue sarcomas [1, 2]. MPNSTs occur either sporadically or in association with neurofibromatosis type 1 (NF1). The overall incidence of MPNST in the general population is 1/100000, of which 5–42% are associated with NF1 [2]. The relative rarity of MPNST and the lack of any specific diagnostic, radiological, or pathological signature pose considerable management challenges for this disease. Even with multidisciplinary treatment, the prognosis for patients with MPNST remains very poor [3]. Therefore, identification of novel prognostic features and therapeutic strategies are required to benefit patients with this aggressive sarcoma.

The fibroblast growth factor (FGF) receptor (FGFR) signaling pathway regulates multiple biological processes, including normal cell growth, survival, differentiation, and angiogenesis. FGFRs are also implicated in tumor development [4, 5]. FGFRs possess an extracellular ligand domain that comprises three immunoglobulin-like domains (I–III), a transmembrane domain, and an intracellular tyrosine kinase domain that transmits signals induced by ligand binding to the interior of the cell [6]. There are four cell-surface FGFRs (FGFR-1–4) and seven FGFR isoforms (1b, 1c, 2b, 2c, 3b, 3c, and 4). These isoforms result from alternative splicing of exons coding for Ig-III-likes domains and account for different ligand-binding specificities [7, 8].

Identification of the roles and relationships within the FGF/FGFR family and of their links with tumor growth and progression is critical for the design of novel drug therapies to target FGFR pathways. FGFR1 inhibitors are considered potential therapeutic agents in *FGFR1*-amplified lung squamous cell carcinoma, and early-stage clinical trials have been conducted [9–11]. Additionally, high expression levels of FGFR1, FGFR2, or FGFR4 are associated with tumor progression and poor survival in patients with gastric cancer [12]. FGFR1 expression in cancer-associated fibroblasts also has prognostic value in head and neck squamous cell carcinoma [13].

We evaluated the prognostic value of FGFR expression in MPNST using a microarray-based comparative genomic hybridization (aCGH) method, fluorescence *in situ* hybridization (FISH), and immunohistochemical (IHC) methods to evaluate the gene status and protein expression levels of FGFR1–4 in MPNST samples. Contrasting with the role of FGFRs in epithelial cancers, high expression of FGFR1 predicted better overall survival (OS) for MPNST patients. Furthermore, combined high expression of FGFR1 and FGFR2 protein characterized a subtype of MPNST with better OS, while increased FGFR4 protein expression predicted worse disease-free survival (DFS).

## RESULTS

### aCGH and FISH detected and validated alterations to *FGFR* genes in MPNST

**Figure 1:**
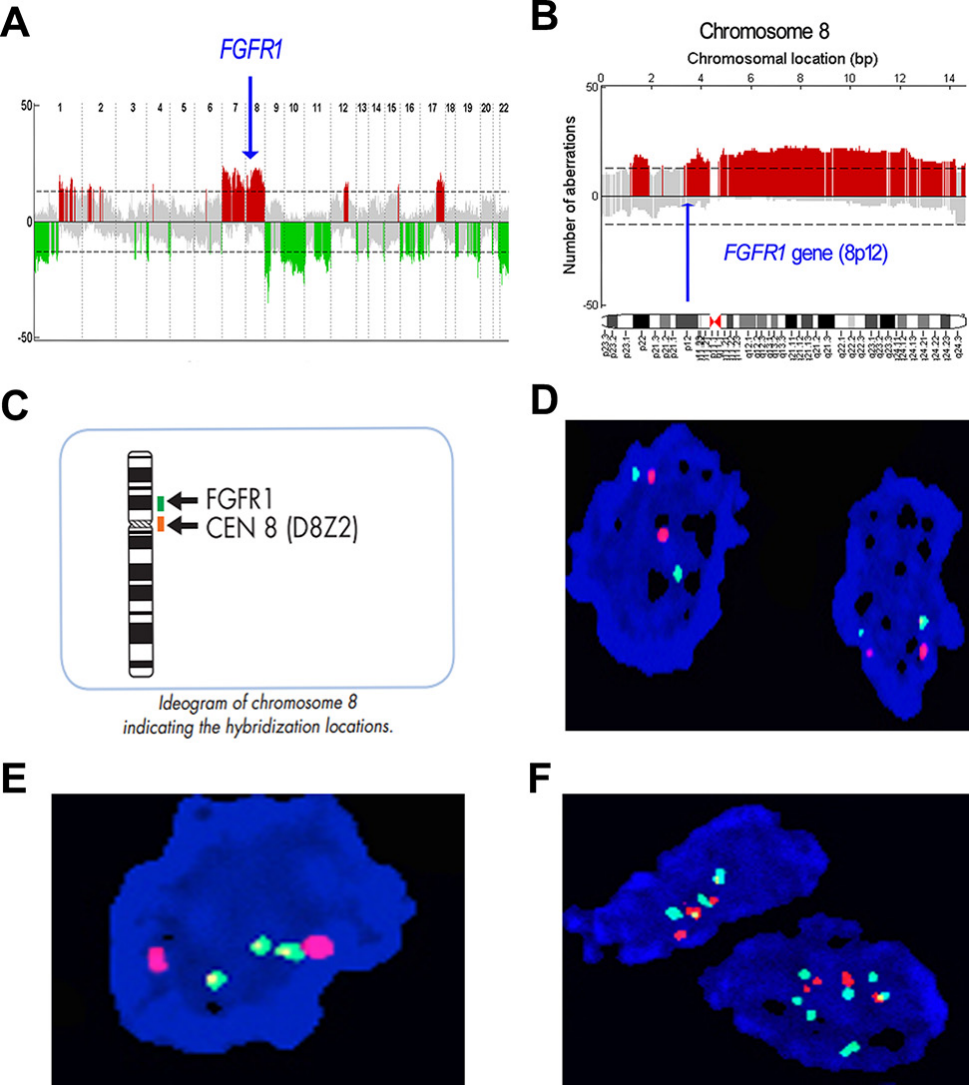
Profile of gene copy number alterations and *FGFR1* gene amplification in MPNST (**A**) Numbers 1–22 on the x-axis denote chromosome numbers. The y-axis indicates recurrence of gains (positive axis) and losses (negative axis) for each measured locus evenly distributed in chromosomal order. Recurrence rates that exceed the threshold (dashed line) are color-coded to emphasize the locations of significantly recurrent aberrations. Red denotes significantly recurrent amplifications and green denotes significantly recurrent deletions. Gray represents non-significant recurrent aberrations. (**B**) Large-fragment amplification of chromosome 8p, including the *FGFR1* gene. Arrow indicates location of the *FGFR1* gene, which is amplified in 37% of cases. (**C**) Schematic depiction of *FGFR1*/Centromere (CEN)-8 Dual Color FISH Probe staining. (**D**) No copy number aberration of FGFR1 gene in MPNST human tissue, green signal represents the centromere and orange signal represents the *FGFR1* gene. (**E**) Focal increase of *FGFR1* gene copy number. (**F**) Increased copy number of the *FGFR1* gene in larger fragment form (polysomy).

Integration of copy number profiles of 51 individual MPNST samples revealed frequent gene deletions and amplifications (Figure 1A; Table 1). Bioinformatics analysis revealed that the amplification rate of the *FGFR1* gene was 37% in MPNST samples (Figure 1B). The deletion rate of the *FGFR2* gene was 41%, while that of *FGFR3* was 27%. There were no significant alterations to *FGFR4*.

**Table 1:** Clinicopathological characteristics of 51 MPNST samples used for aCGH assay

Clinical characteristics	Frequency	Clinical characteristics	Frequency
**Gender**		**Radiotherapy**	
male	29	No	22
female	22	Yes	29
**Age**		**Chemotherapy**	
< 40 year	29	No	26
≥ 40 year	22	Yes	24
**NF1 type**		**Surgical type**	
no	35	radical resection	30
yes	16	subtotal resection	21
**Tumor site**		**Recurrence**	
head and neck	4	No	23
trunk	29	Yes	27
extremity	18	Metastasis	
**Tumor size**		**No**	23
≤ 5 cm	8	Yes	27
5–10 cm	24	**Tumor progression**	
> 10 cm	11	No	19
**AJCC stage**		Recurrence and/or metastasis	31
1	14	**Outcome**	
2	18	Death from tumor	21
3	16	Alive	27
4	20	Lost to follow-up	1

We next validated the *FGFR1* gene amplification findings of the aCGH analysis by conducting FISH analyses on 52 evaluable formalin-fixed paraffin-embedded (FFPE) MPNST samples from Tianjin Medical University Cancer Institute and Hospital (TMUCIH) (Table 2). *FGFR1* probe (green) and centromere (CEN)-8 probe (orange) were co-hybridized to samples on slides (Figure 1C, Figure 1D). Two patterns of *FGFR1* copy number amplification were observed: focal amplification (Figure 1E) and chromosomal arm-level amplification (polysomy) (Figure 1F).

**Table 2:** Clinicopathological characteristics of 52 Chinese MPNST samples used for FISH

Clinical characteristics	Frequency	FGFR1 amplification				Clinical characteristics	Frequency	FGFR1 amplification			
		Yes	No	χ^2^	P			Yes	No	χ^2^	P
**Gender**						**AJCC stage**					
male	27	9	18	1.173	0.279	1	5	1	4	1.425	0.74
female	25	5	20			2	26	6	20		
**Age**						3	7	3	4		
≥ 40 year	32	7	25	1.078	0.299	4	11	3	8		
> 40 year	20	7	13			**Radiotherapy**					
**Age**						yes	20	7	13	0.938	0.333
≥ 30 year	41	11	30	0.001	0.997	no	27	6	21		
< 30 year	11	3	8			**Chemotherapy**					
**NF1 type**						yes	19	6	13	0.322	0.571
no	48	11	37	5.091	**0.024**	no	29	7	22		
yes	4	3	1			**Recurrence**					
**Tumor site**						yes	32	11	21	2.348	0.125
head and neck	7	0	7	5.893	0.053	no	20	3	17		
trunk	21	4	17			**Metastasis**					
extremity	24	10	14			yes	21	6	15	0.049	0.825
**Tumor size**						no	31	8	23		
≤ 5 cm	17	5	12	0.937	0.663						
5–10 cm	21	6	15								
> 10 cm	13	2	11								

We identified *FGFR1* gene copy amplification in 26.9% (14/52) samples. NF1-positive cases had a higher frequency of *FGFR1* gene amplification (χ^2^ = 5.091, *p* = 0.024). *FGFR1* amplification was not correlated with prognosis or any other clinical variables, including gender, age, tumor site, American Joint Committee on Cancer (AJCC) staging, tumor recurrence, or metastasis (Table 2). Furthermore, survival analysis demonstrated that *FGFR1* amplification had no significant impact on DFS (Supplementary Figure S1A) or OS (Supplementary Figure S1B) in this patient cohort.

### High FGFR1 protein expression in MPNST improves OS

**Figure 2:**
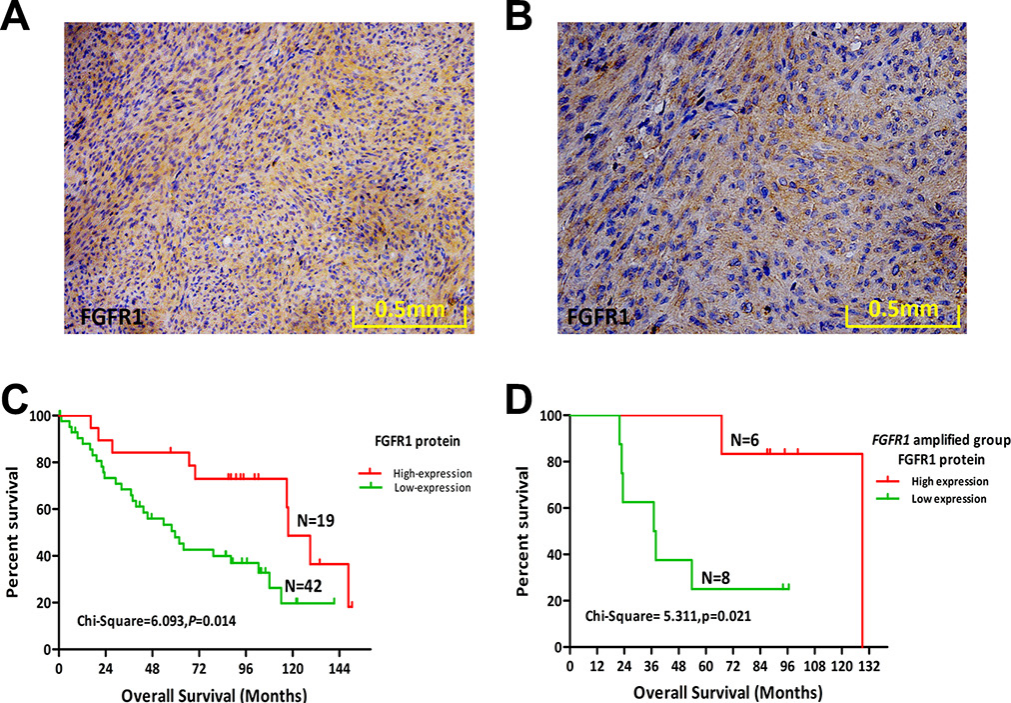
Protein expression levels of FGFR1 and its prognostic role in MPNST (**A**) FGFR1 protein expression in a representative human MPNST tissue sample (20×). (**B**) FGFR1 protein expression in a representative human MPNST tissue sample (40×). (**C**) Kaplan–Meier plot of overall survival (OS) of 61 patients with MPNST based on level of FGFR1 expression. (**D**) Kaplan–Meier plot of OS in the *FGFR1* amplified group (14 cases) of MPNST patients based on FGFR1 expression.

We further examined protein expression of FGFR1 and other FGFR family members by IHC staining of 63 FFPE human MPNST samples (including the 52 samples used for FISH analysis) (Table 3). FGFR1 protein was detected in 30.2% (19/63) of cases (Figure 2A, 2B). *FGFR1* gene amplification and FGFR1 protein expression were positively correlated, suggesting that the increased FGFR1 protein expression partly resulted from *FGFR1* gene amplification (χ^2^ = 4.924, *p* = 0.026; *r* = 0.308, *p* = 0.026).

**Table 3:** Correlation of FGFR1 protein expression with clinicopathological characteristics in 63 MPNST patients

Clinical characteristics	Frequency	FGFR1				Clinical characteristics	Frequency	FGFR1			
		++/+++	–/+	χ^2^	*P*			++/+++	–/+	*χ^2^*	*P*
**Gender**						**AJCC stage**					
male	34	11	23	0.169	0.681	1	7	2	5	0.571	0.948
female	29	8	21			2	32	11	21		
**Age**						3	9	2	7		
≥ 40 year	39	10	29	0.992	0.319	4	12	4	8		
< 40 year	24	9	15			**Radiotherapy**					
**Age**						yes	25	7	18	0.189	0.664
≥ 30 year	49	16	33	0.651	0.42	no	33	11	22		
< 30 year	14	3	11			**Chemotherapy**					
**NF1 type**						yes	25	6	19	0.598	0.439
no	58	17	41	0.25	0.617	no	33	11	22		
yes	5	2	3			**Recurrence**					
**Tumor site**						yes	39	11	28	0.185	0.667
head and neck	10	2	8	4.58	0.101	no	24	8	16		
trunk	26	5	21			**Metastasis**					
extremity	27	12	15			yes	25	7	18	0.092	0.762
**Tumor size**						no	38	12	26		
≤ 5 cm	24	8	16	0.202	0.904								
5–10 cm	22	6	16										
10 cm	16	5	11										

FGFR1 protein expression in MPNST was not correlated with any clinical variables examined (Table 3). Furthermore, FGFR1 protein expression did not affect DFS (Table 4; Supplementary Figure S1C). However, patients with a higher expression of FGFR1 protein had improved OS (χ^2^ = 6.093, *p* = 0.014; Figure 2C; Table 4).

**Table 4:** Prognostic role of FGFRs protein expression in 63 MPNST patients

Group (63 cases)	Disease free survival		Overall survival	
	*χ^2^*	*P*	*χ^2^*	*P*
Gender	.101	.751	.024	.878
Age (≥ 40 year, < 40 year)	.362	.547	.745	.388
NF1 type	16.500	**.000**	6.547	**.011**
Tumor site	1.393	.498	.696	.706
Tumor size (5, 10)	2.466	.291	2.360	.307
AJCC stage	10.110	**.018**	7.418	.060
Radiotherapy	3.346	.067	1.470	.225
Chemotherapy	2.904	.088	1.916	.166
Surgical type	.002	.967	.086	.770
Recurrence	26.550	.000	8.174	**.004**
Metastasis	16.189	.000	8.252	**.004**
FGFR1 (−/+,++/+++)	.456	.500	6.093	**.014**
*FGFR1* FISH (−/+)	.046	.831	.007	.935
FGFR2 (−/+,++,+++)	1.091	.296	.105	.746
FGFR1 combined with FGFR2	.403	.818	6.215	**.045**
FGFR4 (−/+,++/+++)	4.546	**.033**	2.311	.128

Cox proportional hazards analysis revealed NF1 as an independent prognostic predictor of DFS in MPNST (HR = 0.141, 95% CI = 0.045–0.441, *p* = 0.001; Table 5). Meanwhile, recurrence and FGFR1 protein expression were independent prognostic predictors of OS (Table 5). Patients with recurrence had shorter OS (HR = 2.918, 95% CI = 1.215–7.005, *p* = 0.017), while those with high expression of FGFR1 protein had improved OS compared with the low expression group (HR = 0.357, 95% CI = 0.149–0.851, *p* = 0.020).

**Table 5:** Independent predictors of OS and DFS in human MPNST

Disease-free survival	*χ^2^*	*P*	HR	95.0% CI		Overall survival	*χ^2^*	*P*	HR	95.0% CI	
				Lower	Upper					Lower	Upper
NF1(no/yes)	11.34	**.001**	.141	0.0451	0.4409	NF1 (no/yes)	1.918	.166	.475	0.166	1.3615
AJCC	5.779	.123				Recurrence (yes/no)	5.740	**.017**	2.918	1.2151	7.0054
AJCC (1/4)	1.852	.174	.445	0.1384	1.4286	Metastasis (yes/no)	1.767	.184	1.653	0.7879	3.4689
AJCC (2/4)	2.378	.123	.522	0.2285	1.1926	FGFR1 (high/low )	5.402	**.020**	.357	0.1495	0.8508
AJCC (3/4)	4.546	**.033**	.250	0.0698	0.894						
FGFR4 (high/low)	3.305	.069	3.865	0.8997	16.6						

### An MPNST subtype is characterized by *FGFR1* gene amplification, high expression of FGFR1 protein, and improved OS

High expression of FGFR1 protein indicates a poor prognosis for multiple tumor types, including lung cancer, triple-negative breast cancer, and gastric cancer [12, 14, 15]. Contrastingly, we found that high expression of FGFR1 indicated a favorable prognosis for MPNST patients. We further investigated the prognostic role of FGFR1 in MPNST by separating the 52 cases of MPNST samples based upon *FGFR1* gene amplification, forming an amplified group (*n* = 14) and a normal group (*n* = 38). FGFR1 protein expression significantly influenced OS in the *FGFR1*-amplified group (χ^2^ = 5.311, *p* = 0.021; Figure 2D), but had no relationship with DFS (Supplementary Figure S1D). Expression of FGFR1 had no significant effect on DFS or OS in the normal group (Supplementary Figure S1E, S1F). These findings reveal that *FGFR1* gene amplification characterizes a special subtype of MPNST patients with higher expression of FGFR1 protein and improved OS.

### Combined expression of FGFR1 and FGFR2 characterizes a subtype of MPNST patients with better prognosis

**Figure 3:**
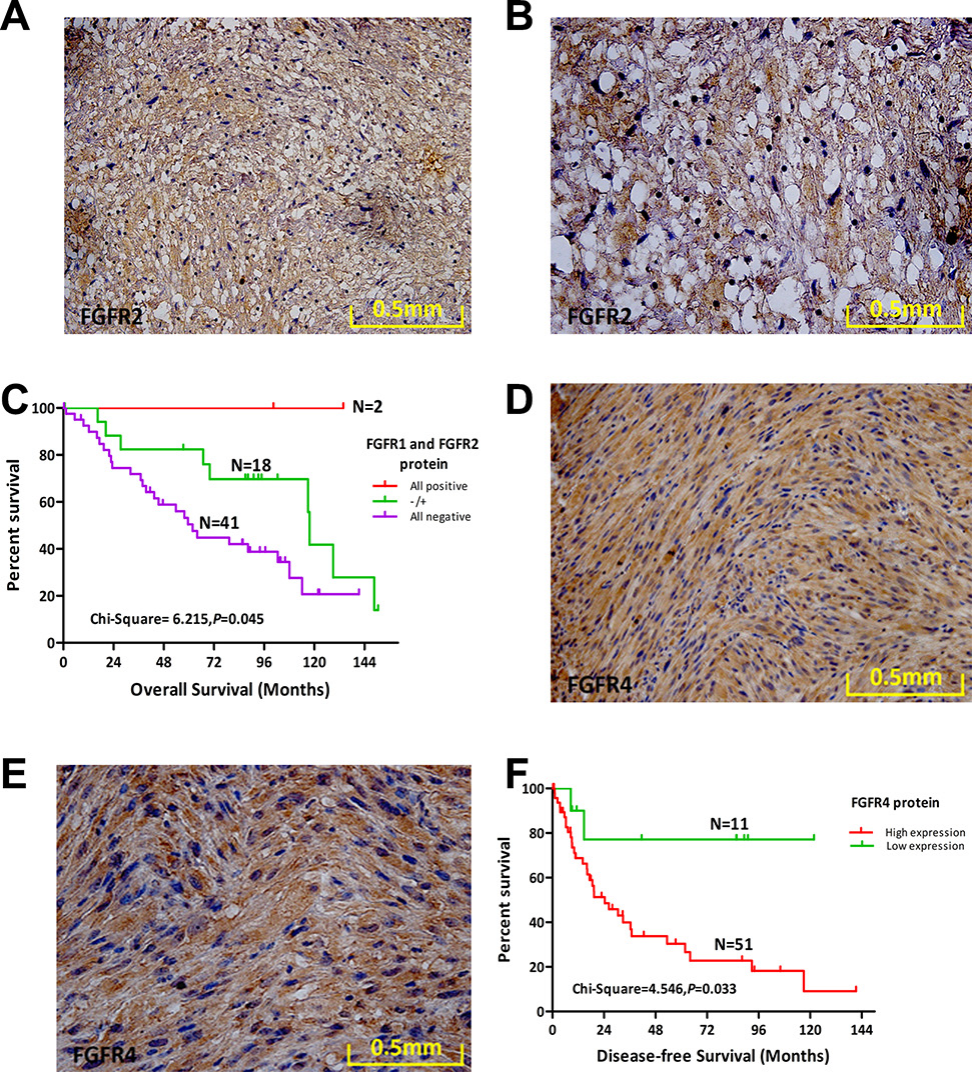
Protein expression levels of FGFR2 and FGFR4 and their prognostic role in MPNST (**A**) FGFR2 protein expression in a representative human MPNST tissue sample (20×). (**B**) FGFR2 protein expression in a representative human MPNST tissue sample (40×). (**C**) Overall survival (OS) of 61 MPNST patients based on FGFR1 and FGFR2 expression status. (**D**) FGFR4 protein expression in a representative human MPNST tissue sample (20×). (**E**) FGFR4 protein expression in a representative human MPNST tissue sample (40×). (**F**) OS of 62 MPNST patients based on FGFR4 expression status.

Our initial aCGH study revealed a deletion rate of 41% for *FGFR2*. We next used IHC to evaluate expression of FGFR2 protein (Figure 3A, 3B). Staining for FGFR2 protein was positive in 19.7% (12/61) of cases, and FGFR2 expression in MPNST was not correlated with DFS, OS (Supplementary Figure S2A, S2B), or any other clinical variables assessed.

We next regrouped patients based on FGFR1 and FGFR2 protein expression as follows: both positive, both negative, or single-positive for either. Patients expressing both FGFR1 and FGFR2 exhibited the best OS (χ^2^ = 6.215, *p* = 0.045; Figure 3C). However, DFS was not significantly different among the three groups (SupplementaryFigure S2C). Combined expression of FGFR1 and FGFR2 enhanced the prognostic value of FGFR1 and FGFR2 in MPNST.

### Increased FGFR4 protein expression predicted worse DFS

Bioinformatics analysis revealed a deletion rate of 27% for *FGFR3* in MPNST. We used IHC to assess expression of FGFR3 protein in 63 MPNST samples. However, compared with the positive FGFR3 protein expression in KMS-11 cells (Supplementary Figure S2D), we did not identify FGFR3 protein expression in any of the 63 MPNST cases (Supplementary Figure S2E).

No significant aberrations in the *FGFR4* gene were detected by aCGH analysis of 51 MPNST tissue samples. However, high levels of FGFR4 protein were present in 82.3% (51/62) of MPNST cases (Figure 3D, 3E). Survival analysis revealed that high expression of FGFR4 conferred worse DFS compared with the low expression group(χ^2^ = 4.546, *p* = 0.033; Figure 3F; Table 4). However, high expression of FGFR4 in MPNST was not correlated with OS or any other clinical variables assessed (Supplementary Figure S2F).

## DISCUSSION

MPNST has been previously known as malignant schwannoma, malignant neurilemmoma, neurogenic sarcoma, and neurofibrosarcoma [16]. MPNST is a rare but highly malignant sarcoma of soft tissues that occurs either sporadically or in association with NF1 [17]. The incredibly poor prognosis for MPNST highlights the need for new prognostic markers in this disease. Additionally, an improved understanding of the key genetic and molecular events involved in MPNST development and progression are critical for the development of effective therapeutics. We conducted genomic and molecular studies of human tumor samples to characterize the roles played by FGFRs in MPNST. Our findings provide evidence of different genetic aberrations among FGFRs in MPNST. Additionally, we demonstrate that FGFRs are potentially useful markers for prognosis of MPNST.

The expression of FGFRs in neurogenic tumors has been poorly studied. Therefore, a major contribution of this study is identification of the genetic status of *FGFR* family member expression in MPNST by aCGH and validation of *FGFR1* gene amplification by FISH. Gene dosage is an important regulator of FGFR protein expression [18, 19]. Consistently, we found that *FGFR1* gene dosage played an important role in increasing FGFR1 protein expression. We also identified deletion of the *FGFR2* and *FGFR3* genes, consistent with the low or negative expression their corresponding proteins. Meanwhile, no significant alterations to the *FGFR4* gene were identified, though FGFR4 protein was expressed at a high level. These findings indicate that overexpression of FGFRs is dependent on both the gene copy number and other mechanisms such as aberrant transcriptional regulation.

Additionally, internalization and degradation of the activated FGF–FGFR complex is an important consideration [5]. The four FGFRs are ubiquitinated to different extents, and this ubiquitination appears to dictate whether these receptors are transported back to the plasma membrane or degraded. FGFR1 generally undergoes extensive ubiquitination and is transported to lysosomes for degradation. Meanwhile, FGFR4 is only lightly ubiquitinated, inefficiently degraded, and is preferentially recycled to the cell surface. Therefore, further evaluation of the mechanisms underlying the differential regulation of FGFR family members is important for a more complete understanding of the precise roles of FGFRs in tumor development.

We identified improved prognosis for MPNST patients with higher expression of FGFR1. Additionally, FGFR1 protein expression is an independent prognostic predictor of improved OS. However, FGFR1 expression is associated with unfavorable outcomes in other tumors, including lung cancer, triple-negative breast cancer, and gastric cancer, and FGFR1 may be a useful therapeutic target in these diseases [12, 14, 15]. Shin et al. identified upregulation of FGFR1, FGFR2, and FGFR4—but not FGFR3—in gastric cancer tissues [20]. Another study identified high expression of all four FGFRs in gastric cancer tissues [12]. Additionally, a reciprocal relationship between FGFR1 and FGFR3 in colorectal tissue has been characterized [21]. These apparent discrepancies in FGFR expression among studies may be attributable to differences in disease stage or the techniques used for protein expression analysis.

Importantly, although FGF signaling can promote tumorigenesis, in certain contexts FGFRs can also mediate anti-tumor functions. FGFR expression can be down-regulated though autocrine or paracrine FGF–FGFR signaling loops out-of-context, indicating that FGFRs may act as tumor suppressors in certain contexts [5, 21]. For example, if an FGFR-expressing cell also overexpresses the corresponding ligand, an autocrine loop can be established and the cancer cell becomes self-sufficient in growth signals.

FGFRs play important roles in tumor progression by activating downstream signaling pathways. Two classes of regulator modulate signaling output from activated FGFRs. Negative regulators include Sprouty proteins, mitogen-activated protein kinase phosphatase 3(MKP3), and similar expression to FGF protein [22, 25] Positive regulators of FGFR signaling include the fibronectin-leucine-rich transmembrane proteins (FLRT)-1–3 [26, 27]. Therefore, high expression of FGFR1 may simply serve as a marker of favorable prognosis rather than being the actual mechanism underlying the favorable prognostic outcome for these MPNST patients.

Our data reveal that the different FGFRs have different prognostic roles for MPNST. FGFR1 expression was an independent predictor of favorable OS, while FGFR4 protein expression predicted poor prognosis. These differences may reflect the differing physiological functions of FGFR1 and FGFR4. Consistently, the different FGFRs play an assortment of roles in numerous tumors and tissues, and co-expression of FGFRs in various combinations may cause subtle changes in the progression of cancer [28, 29]. Contrasting with the role of FGFRs in epithelial cancers, FGFR1 and FGFR2 protein expression characterized a subtype of MPNST with better OS. Additionally, overexpression of FGFR4 is associated with advanced stage cancer and poor survival in rhabdomyosarcoma (RMS). FGFR4 protein is expressed in the two main variants of RMS—embryonal RMS and alveolar RMS (aRMS). Interestingly, only certain subgroups of aRMS cells are rescued by FGFR4 signaling following induction of apoptosis by compounds targeting the IGF1R-PI3K-mTOR pathway [30–32]. Therefore, expression patterns of FGFRs could facilitate selection of patients for adjuvant systemic therapy. Importantly, as this effect is seen at the protein level, the biomarker panel can be readily implemented in routine clinical testing using IHC.

We used integrated genetic and molecular profiling to confirm genetic alterations of the *FGFR1–4* genes and measure expression of the corresponding proteins in MPNST tissues. High expression of FGFR1 was an independent prognostic predictor of OS, and tumors with higher expression of FGFR1 had better prognosis than those with lower expression. Additionally, patients with tumors expressing high levels of FGFR4 had worse DFS. However, the molecular details underlying the observed effects are largely unknown. Additional thorough investigations and clinical trials are needed to enhance our understanding of the different prognostic roles for FGFR family members in MPNST.

## MATERIALS AND METHODS

### Patients and primary tumors

Patient information collected included age, sex, NF1 status, tumor location, largest diameter of the tumor, clinical AJCC stage, time to recurrence, metastatic status, treatments, and outcome. The presence of the NF1 syndrome was determined by the NIH criteria. Collection of tissue and information for this retrospective study was approved by the Institutional Review Boards at Tianjin Medical University Cancer Institute and Hospital and The University of Texas, MD Anderson Cancer Center, and occurred with patient consent [33, 34].

A cohort of 51 patients with histologically confirmed MPNST and matching patient records was included in this study for genome-wide copy number measurements using the aCGH method [33, 34]. Of these samples, 25 FFPE tumor specimens were acquired from The University of Texas MD Anderson Cancer Center, and another 26 fresh tumor samples were from the Tianjin Cancer Hospital of China [33, 34]. IHC validation was performed using an independent cohort of 63 FFPE tumor samples acquired from TMUCIH, with 52 of these FFPE tumor samples subjected to FISH for validation of specific copy number aberrations.

### Array CGH hybridization and bioinformatic analysis

Genome-wide copy number levels for the 51 primary tumor samples were mapped using aCGH with commercially available normal genomic DNA as a reference (Clontech Laboratories, Inc., Mountain View, CA) [33]. Genomic DNA was isolated from tumors according to standard procedures and the labeled genomic DNAs were hybridized using the Agilent 4 × 44 k Human Genome CGH Microarray kit (Agilent Technologies, Santa Clara, CA). Processing of aCGH data and frequency analyses were performed as described previously [33, 34]. Briefly, the ratios of intensity values from tumor and normal tissues were transformed to log2-space. Log ratio data were then subjected to a circular binary segmentation algorithm to reduce the effect of noise. After that, the CGHcall algorithm was used to give each segment an aberration label: normal, deletion, or amplification. An aberration frequency for each probe was established by combining the labels from individual samples.

### FISH analysis

The ZytoLight SPEC *FGFR1/CEN 8* Dual Color Probe (ZytoVision, Bremerhaven, Germany) was used for detection of *FGFR1* gene amplification. The CEN8 probe exhibits an orange signal and indicates the centromere of chromosome 8, while the *FGFR1* probe exhibits a green signal. FISH was performed as previously described [35]. Briefly, deparaffinized sections were pretreated by incubation with deionized water at 95°C for 20 min followed by digestion with pepsin at 37°C for 50 min. Tissue sections and *FGFR1*/CEN8 FISH probes were then denatured at 78°C for 10 min and incubated overnight at 37°C to allow hybridization. Slides were then washed and samples counterstained with 4′, 6-diamidino-2-phenylindole and mounted with coverslips.

Alterations of *FGFR1* gene copy number were evaluated by two pathologists in a blinded fashion according to established methods [36–38]. An increase in *FGFR1* gene copy number was defined as an FGFR1/CEN8 signal ratio ≥ 1.5 and when two or more *FGFR1* gene copies were present per cell in > 90% of MPNST cells [33].

### Immunohistochemical analysis

IHC analysis was employed to evaluate FGFR protein expression. Antibodies used were anti-FGFR1 (Cell Signaling Technology; 1:500), anti-FGFR2 (R & D Systems; 22 μg/mL), anti-FGFR3 (Cell Signaling Technology; 1:50), and anti-FGFR4 (R & D Systems; 15 μg/mL). PBS was used as negative control. Microscopically, ten high-power (40×) fields from each section were observed randomly and 100 cells were scored. Staining was semi-quantified into four scores based upon the number of positive cells: < 5% (score 0), 5–25% (score 1), 26–50% (score 2), 51–75% (score 3), and > 75% (score 4). Staining intensity followed a four-score classification: no cell stain (score 0), yellow (score 1), tan (score 2) and brown (score 3). Final IHC scores were calculated by addition of intensity and extent scores, and the results were divided as follows: negative (−; scores 0 and 1), weakly positive (+; scores 2 and 3), moderately positive (++; scores 3–5), and strongly positive (+++; scores 6 and 7). Results of staining for FGFR1–4 were organized into low expression (negative and weakly positive) and high expression (moderately and strongly positive) groups [28].

### Statistical analysis

Correlations between FGFR expression and clinicopathological variables were analyzed using the Chi-square test. Patient survival curves were plotted according to the Kaplan–Meier method and a log-rank test. Multivariate Cox regression analysis was used to identify significant independent prognostic factors. OS was defined as the time period from the date of diagnosis to death or the last follow-up. For DFS analysis, the duration was defined as the time from diagnosis until the occurrence of metastasis or recurrence. A two-sided *p*-value < 0.05 was considered statistically significant. All statistical analyses were performed using SPSS version 19.0 statistical software (SPSS, Chicago, IL).

## SUPPLEMENTARY MATERIAL FIGURES


