# Supplementary material for: Prognostic roles for fibroblast growth factor receptor family members in malignant peripheral nerve sheath tumor
Source: Oncotarget. 2016 Mar 14;7(16):22234–44. doi: 10.18632/oncotarget.8067 (PMC5008358; doi:10.18632/oncotarget.8067)
Supplement: Supplementary file 1 [file oncotarget-07-22234-s001.pdf]

Prognostic roles for fibroblast growth factor receptor family members in malignant peripheral nerve sheath tumor

Supplementary Materials

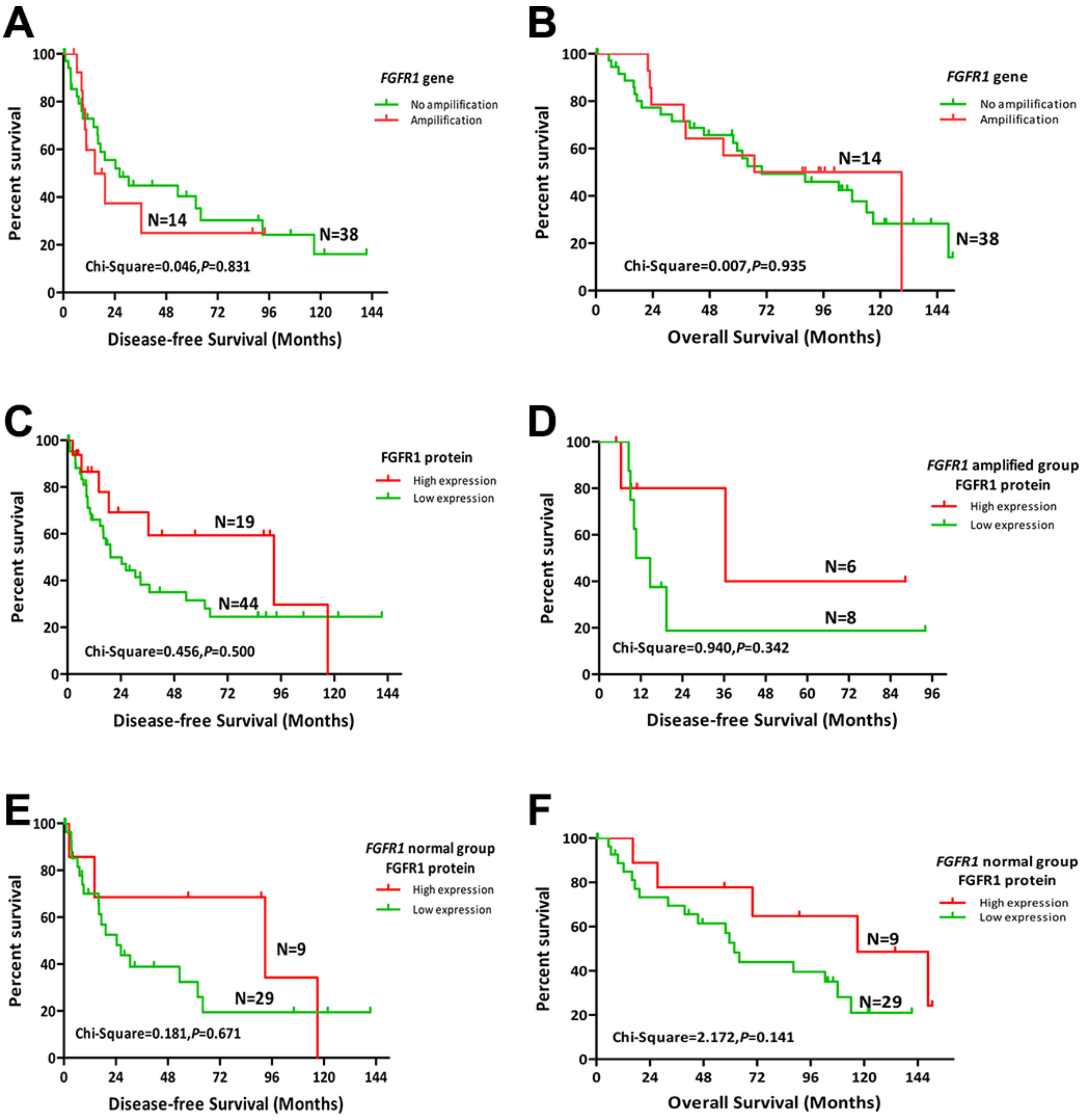

Supplementary Figure S1: Survival analysis based on *FGFR1* gene status and FGFR1 protein expression level. (**A**) Kaplan–Meier plot of disease-free survival (DFS) of 52 patients with MPNST based on status of *FGFR1* gene expression. (**B**) Kaplan–Meier plot of overall survival (OS) of 52 patients with MPNST based on status of *FGFR1* gene expression. (**C**) Kaplan–Meier plot of DFS of 63 patients with MPNST based on level of FGFR1 protein expression. (**D**) Kaplan–Meier plot of DFS in the *FGFR1*-amplified group (14 cases) of MPNST patients based on FGFR1 protein expression. (**E**) Kaplan–Meier plot of DFS in the *FGFR1*-normal group (38 cases) of MPNST patients based on FGFR1 protein expression. (**F**) Kaplan–Meier plot of OS in the *FGFR1*-normal group (38 cases) of MPNST patients based on FGFR1 protein expression.

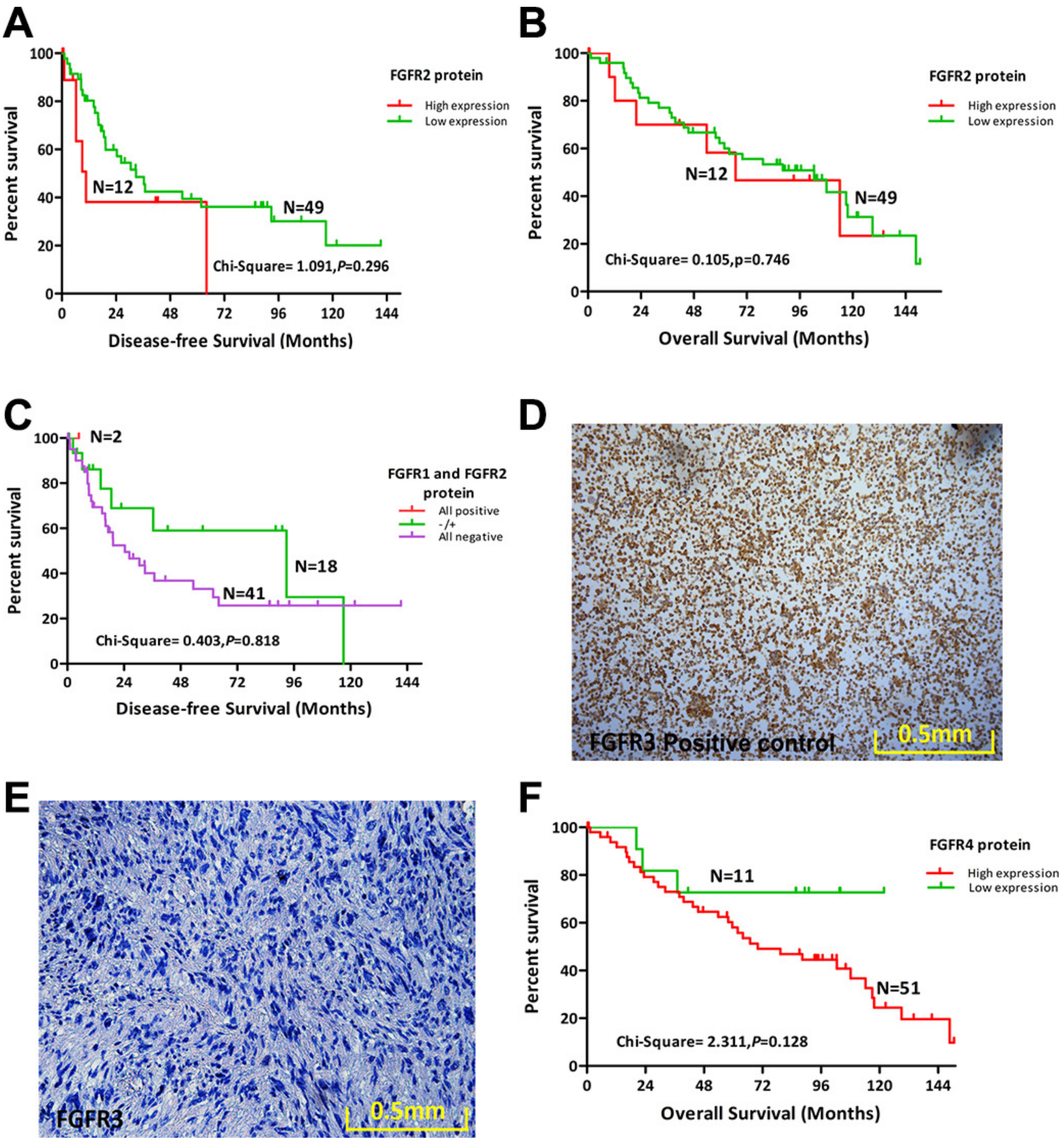

Supplementary Figure S2: Survival analysis based on FGFR2, FGFR1 and FGFR2, and FGFR4 protein expression levels. (**A**) Kaplan–Meier plot of disease-free survival (DFS) of 61 patients with MPNST based on level of FGFR2 protein expression. (**B**) Kaplan–Meier plot of overall survival (OS) of 61 patients with MPNST based on level of FGFR2 protein expression. (**C**) DFS of 59 MPNST patients based on FGFR1 and FGFR2 protein expression status. (**D**) FGFR3 protein expression in KMS-11 cells (positive control) (40×). (**E**) FGFR3 protein expression in a representative human MPNST tissue sample (40×). (**F**) Kaplan–Meier plot of OS of 62 patients with MPNST based on level of FGFR4 protein expression.
